# Vaccination of Koalas with a Recombinant *Chlamydia* *pecorum* Major Outer Membrane Protein Induces Antibodies of Different Specificity Compared to Those Following a Natural Live Infection

**DOI:** 10.1371/journal.pone.0074808

**Published:** 2013-09-25

**Authors:** Avinash Kollipara, Adam Polkinghorne, Kenneth W. Beagley, Peter Timms

**Affiliations:** Institute of Health and Biomedical Innovation, Queensland University of Technology, Kelvin Grove, Queensland, Australia; University of Lausanne, Switzerland

## Abstract

Chlamydial infection in koalas is common across the east coast of Australia and causes significant morbidity, infertility and mortality. An effective vaccine to prevent the adverse consequences of chlamydial infections in koalas (particularly blindness and infertility in females) would provide an important management tool to prevent further population decline of this species. An important step towards developing a vaccine in koalas is to understand the host immune response to chlamydial infection. In this study, we used the Pepscan methodology to identify B cell epitopes across the Major Outer Membrane Protein (MOMP) of four *C* *. pecorum* strains/genotypes that are recognized, either following (a) natural live infection or (b) administration of a recombinant MOMP vaccine. Plasma antibodies from the koalas naturally infected with a *C* *. pecorum* G genotype strain recognised the epitopes located in the variable domain (VD) four of MOMP G and also VD4 of MOMP H. By comparison, plasma antibodies from an animal infected with a *C* *. pecorum* F genotype strain recognised epitopes in VD1, 2 and 4 of MOMP F, but not from other genotype MOMPs. When *Chlamydia-*free koalas were immunised with recombinant MOMP protein they produced antibodies not only against epitopes in the VDs but also in conserved domains of MOMP. Naturally infected koalas immunised with recombinant MOMP protein also produced antibodies against epitopes in the conserved domains. This work paves the way for further refinement of a MOMP-based *Chlamydia* vaccine that will offer wide cross-protection against the variety of chlamydial infections circulating in wild koala populations.

## Introduction

The koala is the only surviving member of the family *Phascolarctidae* and is considered an icon of Australia’s unique biodiversity. Despite this esteem, wild koala populations in geographically diverse regions throughout the country continue to decline. This decline has been attributed to several variables such as (a) habitat loss, resulting in fragmentation of koala colonies [1]; (b) disease [2]; (c) motor vehicle trauma [3]; and (d) dog attacks [4]. A recent study showed that addressing disease, amongst the many variables affecting koala survival would have the greatest potential impact on stabilising population decline [2]. Disease caused by infections of the obligate intracellular bacterial pathogen, *Chlamydia* *pecorum* , is a major threat to the ongoing survival of this species [5]. *C* *. pecorum* infections in koalas have been associated with a spectrum of diseases ranging from keratoconjunctivitis (ocular disease) leading to blindness, rhinitis and pneumonia, as well as urinary and genital tract disease, resulting in inflammation and fibrosis of the bladder and the upper female genital tract [6-10]. An effective vaccine to prevent the complications of chlamydial infections in koalas would provide a valuable management tool to stop the decline in wild populations by (a) reducing the infectious load in infected animals, and (b) preventing the further development of chlamydial pathology in healthy animals and development of pathology in already infected animals.

An ideal chlamydial vaccine should be able to induce both cellular and humoral immune responses in the host [11]. The Major Outer Membrane Protein (MOMP), which constitutes 60% of the chlamydial outer membrane, has been the most widely used antigen either in its native or recombinant form in several vaccine studies [12-15]. Initial efforts to develop a MOMP-based vaccine demonstrated a vaccine induced cell-mediated immune response lasting for more than a year as well as a humoral immune response ( *Chlamydia* -neutralizing antibody) lasting > 35 weeks [16]. Following this study, our group further evaluated the safety and efficacy of a *C* *. pecorum* MOMP-based multi-subunit vaccine in diseased as well as healthy koalas [17]. Strong antibody (including neutralizing antibodies) and lymphocyte proliferation responses were recorded in all vaccinated healthy and clinically diseased koalas. Vaccine induced antibodies specific for MOMP G, one of the thirteen known *C* *. pecorum* *ompA* genotypes (A-H; unpublished data) were observed not only in plasma but also in ocular secretions.

In the most recent study, we evaluated the immunogenicity of a vaccine consisting of either monovalent or polyvalent MOMPs [18]. Animals immunized with individual MOMPs developed strong antibody and lymphocyte proliferation responses to both homologous as well as heterologous MOMP proteins. Importantly, we also showed that vaccine-induced antibodies effectively neutralized heterologous strains of koala *C* *. pecorum* in an *in vitro* assay. Finally, we also demonstrated that the immune responses in monovalent as well as polyvalent MOMP vaccine groups were able to recognize whole chlamydial elementary bodies, illustrating the feasibility of developing an effective MOMP-based *C* *. pecorum* vaccine that could protect against a range of strains. A promising aspect of our most recent trials [17,18] was the cross-reactivity of MOMP antibody responses from vaccinated healthy and diseased koalas, giving hope for the generation of a MOMP-based vaccine that will offer wide cross-protection against the variety of genetically distinct *C* *. pecorum* strains circulating in wild koala populations. In the present study, we further investigated the MOMP B cell epitopes responsible for the cross reactivity of the vaccine induced plasma antibodies in our previous vaccine trials. We examined (a) the specific MOMP epitopes that were recognized by koalas naturally infected with *C* *. pecorum* , (b) epitopes recognized by healthy animals immunized with either MOMP A, MOMP F, MOMP G or MOMP A plus F, and (c) epitopes recognized by the diseased animals immunized with MOMP G.

## Materials and Methods

### Koalas used in this study

Nine koalas were analysed as part of this study, consisting of (a) five wild animals with a current or recent *C* *. pecorum* infection and overt signs of disease at the time of sampling, and (b) four captive healthy animals, with no evidence of infection or disease (Table 1). Among the diseased animals, three koalas were tested and found to be infected with *C* *. pecorum* *omp*A genotype F and the remaining two koalas were tested and found to be infected with *C* *. pecorum* *omp*A genotype G [17]. One koala (Popeye) in the *C* *. pecorum* G group and two koalas (Nixon/Felix Pitt) in the *C* *. pecorum* F group were subcutaneously immunized with a vaccine consisting of MOMP G and ISC (adjuvant), as previously described [17]. Kathy received the placebo (adjuvant only). Four healthy animals with no signs of infection or disease were immunized with individual MOMP types (A, F and G) or together (A and F), respectively [18].

**Table 1:** List of diseased as well as healthy koalas in this study with their qPCR status, infecting strain and clinical disease observed during the study.

**Koala Identifier**	** *Chlamydia* PCR status**	**Naturally infected with *C* *. pecorum* (strain**)	**Clinical disease status**	**Immunization**
Mars Bar	+	*C* *. pecorum* G	Cystitis	Not Immunised
Popeye [19]	+	*C* *. pecorum* G	Conjunctivitis	MOMP G
Nixon [19]	+	*C* *. pecorum* F	Conjunctivitis	MOMP G
Felix Pitt [19]	+	*C* *. pecorum* F	Conjunctivitis	MOMP G
Kathy [19]	+	*C* *. pecorum* F	Cystitis	Placebo only
Amity [20]	-	-	-	MOMP A
Nessie [20]	-	-	-	MOMP F
Jaffa [20]	-	-	-	MOMP G
Guppy [20]	-	-	-	MOMP A + MOMP F

References are listed in the brackets.

The sampling and analysis of samples from wild koalas received for treatment at Australian Zoo Wildlife Hospital was performed under permission of the Queensland State Government Department of Environment and Heritage Protection (Scientific Purposes Permit WISP 06056009). All animal work was approved by the Queensland University of Technology Animal Ethics Committee (Approval #0700000845 & #0700000559).

### Sampling of the animals

Animals were sampled prior to immunisation (all 9 animals) and then 20 weeks post immunisation (8 out of 9 animals). Plasma was separated from anti-coagulated bloods by centrifugation at 233x*g* for 5 min at 4°C. Samples were then stored at -80°C for further analysis.

### Screening for *C* *. pecorum* infections

Ocular and urogenital tract swab samples from all animals, prior to immunisation were screened for the presence of *C* *. pecorum* infections by 16S rRNA species-specific quantitative PCR [19] and for the presence of *C* *. pecorum* MOMP specific antibodies by immunoblotting [17].

### Design of Biotinylated peptide library for screening MOMP antibodies from koala plasma

**Figure 1:**
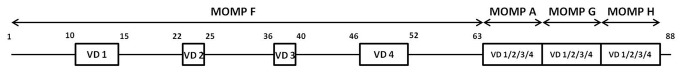
Layout of *C* *. pecorum* MOMP peptide library. Peptides 1 to 63 represent five conserved domains (straight lines) interspersed by four variable domains (boxes) of MOMP F. Peptides 64 to 88 represent four variable domains of MOMP A, MOMP G and MOMP H, respectively.

A library of overlapping 15 amino acid peptides were designed by our group and constructed by Mimotopes (Melbourne, Australia). The full-length koala *C* *. pecorum* MOMP sequence for MOMP F (Figure 1) was used to design the first 63 overlapping (9 amino acid offset) 15 mer peptides. In addition to peptides representing the complete 370 amino acids of *C* *. pecorum* MOMP F, the four variable domain regions of *C* *. pecorum* MOMPs A, G and H, and three peptides representing two major predicted B cell epitopes in conserved domains were also covered by the remaining 25 overlapping 15mer peptides. Each peptide consisted of a biotin molecule linked to a spacer (-SGSG-) to prevent steric hindrance, followed by the 15mer sequence with an offset of 9 amino acids.

### Biotinylated Peptide Enzyme Linked ImmunoSorbent Assay

The wells of 96 well streptavidin coated plates pre-blocked with BSA (Thermo, Fisher Scientific, Melbourne, Australia) were coated individually with each of the 88 biotinylated peptides at a concentration of 2 µg/well in 1X Phosphate Buffered Saline Tween-20 and incubated for 2 hrs at room temperature (22°C). Post incubation, the wells were washed 3X with PBS-T and the individual plasma samples (diluted to 1/1000) were added to the wells (100 µl/well), respectively. The plates were incubated overnight at 4°C followed by washing 4X with PBS-T. The plates were then incubated with sheep anti-koala IgG (1:4000 in PBS-T) (100 µl/well) and incubated for 1 hr at room temperature. Finally, after four washes, HRP-labelled rabbit anti-sheep IgG (1:1000 Southern Biotech/In vitro Technologies, Cleveland, Australia) was added (100 µl/well) to the wells and incubated for 1 hr at room temperature. After five washes with 1X PBS, 100 µl/well of ABTS

[2, 2’-azino-bis (3-ethylbenzothiazoline-6-sulphonic acid), Southern Biotech, Alabama, USA] substrate solution was added and incubated for 10 min at room temperature. The optical density was then read at 405 nm (Bio-Rad, North Ryde, Australia). Background for each sample was calculated from the mean plus twice the standard deviation of the negative wells (no sera added). In an attempt to minimise the inter-assay variability and truly reflect the differences in antibody response profiles between samples, (a) the assay was performed on both pre- and post-immunised samples of each animal alongside on the same day, and (b) the background absorbance (green line) used for comparing the response profiles from pre- and post-immunisations was a result of the mean plus two times the standard deviation of the negative controls of both samples.

### Western blot

Western blots to assess the specificity of the koala plasma to different *C* *. pecorum* MOMP proteins was performed as previously described [17]. In brief, 5 µg aliquots of each of the MOMP’s A, F, G and H was loaded onto 12% SDS-PAGE gels and run at 110V for 1hr. Following blotting onto a nitrocellulose membrane at 90V for 1hr, membranes were blocked in skim milk solution for 1hr. Plasma samples from 4 weeks prior to immunization and 20 weeks post immunization (1:1000) were then analysed by incubating onto respective blots overnight at 4°C. Membranes were then washed and sheep anti koala IgG was added at 1:1000 dilution and left for an hour. After incubation, membranes were further washed with TBST and incubated with HRP-labelled rabbit anti-sheep IgG (1:1000) for 1hr. Blots were then washed thoroughly with TBS and visualised for bands by adding chemiluminescence substrate.

## Results

### Response profile of plasma from *Chlamydia* negative koalas

**Figure 2:**
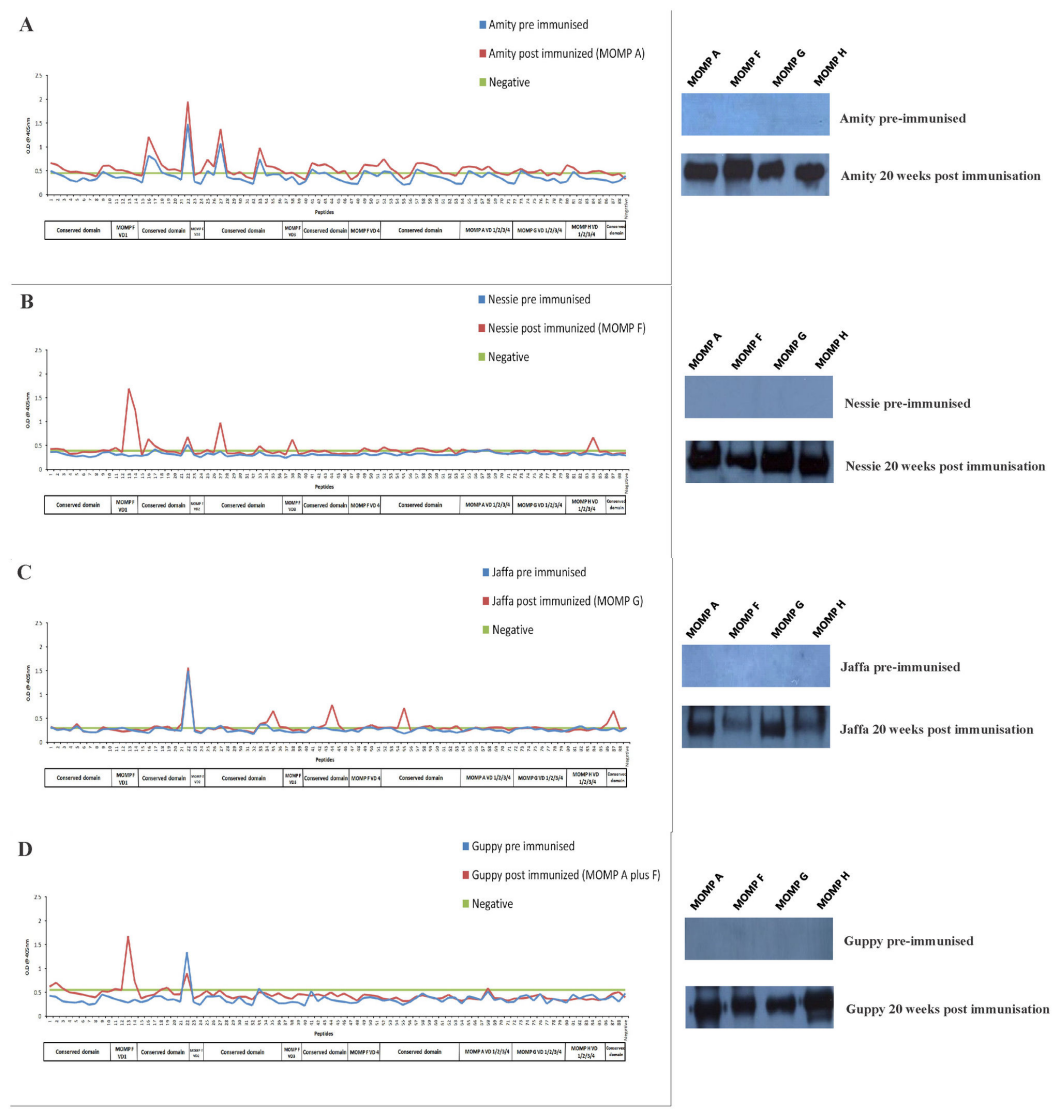
B cell epitope mapping across four MOMPs in *Chlamydia* free koalas after vaccination. Response profiles and immunoblots of four *Chlamydia* free koalas vaccinated with MOMP A (**A**), MOMP F (**B**), MOMP G (**C**) and MOMP A as well as MOMP F (**D**), respectively. The blue line indicates the absorbance of pre immunized samples collected from individual *Chlamydia* -negative animals. The red line indicates the 20 weeks post immunized sample. The green line indicates the background (Mean and two times standard deviation of negative controls).

Plasma from four healthy, pre-immunised, *Chlamydia* negative koalas (Amity, Nessie, Jaffa and Guppy) were evaluated against the 88 peptides in the MOMP peptide library. Figure 2 shows each individual animal’s response (indicated by the blue lines; A, B, C and D). There were weak absorbance levels to all 88 peptides (except for peptide 22 in the conserved domain) in all four healthy, *Chlamydia* -free animals. The response profiles for each pre-immunisation sample was used to establish a background response profile for analysis of post-immunisation samples.

### Vaccination elicits cross-reactive MOMP antibodies in *Chlamydia* free animals immunized with recombinant MOMP proteins A, F and G

Figure 2A shows the specificity of the antibodies produced by a *Chlamydia* negative koala (Amity) following immunization with recombinant MOMP A. Interestingly, when compared to animals immunised with other MOMPs (Figure 2), Amity’s post immunised sample had a better (but not extremely high) response profile against the majority of the same peptides recognized by her own pre-immunised sample. Immunization also induced cross-reactive antibody against three other MOMPs, as shown in the immunoblots of Figure 2A. Another *Chlamydia* negative koala (Nessie) was immunized with recombinant MOMP F protein and by 20 weeks post vaccination produced plasma antibodies (Figure 2B) that reacted strongly against epitopes in (a) the conserved domains (peptides 16 and 27), (b) variable domains of MOMP F (peptides 13, 14 and 38) and; (c) variable domain two of MOMP H (peptide 84) (Table 2). Immunisation of a *Chlamydia* negative animal (Jaffa) with MOMP G protein resulted in antibodies that primarily recognise various linear epitopes in the conserved domains of MOMP (peptides 35, 44, 55 and 87) (Figure 2C). Finally, when a *Chlamydia* negative animal was immunised with a combination of two MOMP proteins (A plus F) it produced antibodies that recognised the epitopes in conserved domains (peptides 2 and 19) as well as the variable domain one of MOMP F (peptides 13 and 14) (Figure 2D) (Table 2). Both the animals immunised with MOMP F (one in combination with MOMP A) gave the strongest reaction to peptide 13 (DSVSLQERENPAYGK) and its overlapping peptide 14 (ERENPAYGKHMHDAE). Additionally, to corroborate the Pepscan data, we performed immuno-blot assays on both pre- and post-immunised plasma at the same time points, using similar concentrations of plasma (1:1000) used in Pepscan assay for each animal. Although the pre-immunised samples were negative against four MOMP variants, the samples collected 20 weeks after immunisation developed cross-reacting antibodies to various MOMPs (Figure 2)

**Table 2:** List of all major epitopes recognised by *Chlamydia* free, healthy animals; *C* *. pecorum* infected koalas; *Chlamydia* free, healthy koalas upon vaccination and *C* *. pecorum* infected koalas upon vaccination.

			** *C* *. pecorum* negative koalas**	** *C* *. pecorum* G infected koalas**		** *C* *. pecorum* F infected koalas**		** *C* *. pecorum* free vaccinated koalas**				** *C* *. pecorum* infected vaccinated koalas**			
**Peptide No.**	**MOMP region**	**Peptide Sequence**	**Mean of four *C* *. pecorum* free pre-immunised animals**	Mars Bar*	Popeye*^#^	Nixon*	Felix Pitt* ^$^	**MOMP A immunised@**	**MOMP F immunized @**	**MOMP G immunised@**	**MOMP A plus MOMP F immunised@**	**Kathy prior to placebo administration ^**	**Kathy 20 weeks post placebo administration ^**	**Popeye vaccinated with MOMP G^#^**	**Felix Pitt vaccinated with MOMP G ^$^**
1		**LPVGNPAEPSLLIDG**													
2		**AEPSLLIDGTIWEGM**									**EI, Post**				
3		**IDGTIWEGMSGDPCD**													
4		**EGMSGDPCDPCATWC**													
5	Conserved domain	**PCDPCATWCDAISLR**													
6		**TWCDAISLRVGFYGD**													
7		**SLRVGFYGDYVFDRV**													
8		**YGDYVFDRVLKTDVP**													
9		**DRVLKTDVPQKFSMG**													
10		**DVPQKFSMGQAPSTN**													
11	*C* *. pecorum*	**SMGQAPSTNSPADSV**													
12	MOMP F	**STNSPADSVSLQERE**													
13	Variable	**DSVSLQERENPAYGK**							**EI, Post**		**EI, Post**	**EIPre**	**EIPre**		
14	Domain 1	**ERENPAYGKHMHDAE**							**EI, Post**		**EI, Post**				
15		**YGKHMHDAEWFTNAG**													
16		**DAEWFTNAGYIALNI**	**EIPre**	**EIPre**	**EIPre**		**EIPre**	**EI, Post**	**EI, Post**						
17	Conserved domain	**NAGYIALNIWDRFDV**													
18		**LNIWDRFDVFCTLGA**													
19		**FDVFCTLGATSGYFK**									**EI, Post**				
20		**LGATSGYFKGNSSSF**													
21		**YFKGNSSSFNLIGLI**													
22		**SSFNLIGLIGISGSD**	**EIPre**	**EIPre**								**EIPre**	**EIPre**	**EI, Post**	**EI, Post**
23	*C* *. pecorum* MOMP F	**GLIGISGSDLNSKVP**				**EIN**	**EIN**								
24	Variable domain 2	**GSDLNSKVPNASISN**													
25		**KVPNASISNGVVELY**						**EI, Post**							
26		**ISNGVVELYTDTTFS**													
27		**ELYTDTTFSWSVGAR**	**EIPre**	**EIPre**		**EIPre**			**EI, Post**						
28		**TFSWSVGARGALWEC**													
29		**GARGALWECGCATLG**													
30	Conserved domain	**WECGCATLGAEFQYA**													
31		**TLGAEFQYAQSKPRV**													
32		**QYAQSKPRVQELNVL**													
33		**PRVQELNVLSNVAQF**	**EIPre**			**EIPre**								**EI, Post**	
34		**NVLSNVAQFTVHRPK**													
35		**AQFTVHRPKGYVNQT**								**EI, Post**					
36		**RPKGYVNQTLPLPIT**													
37	*C* *. pecorum* MOMP F	**NQTLPLPITAGTATD**										**EIPre**	**EIPre**		
38	Variable Domain 3	**PITAGTATDSNEKLK**							**EI, Post**						
39		**ATDSNEKLKNATINY**													
40		**KLKNATINYHEWQVG**													
41		**INYHEWQVGAALSYR**	**EIPre**												
42		**QVGAALSYRLNMLIP**													
43	Conserved domain	**SYRLNMLIPYIGVQW**													
44		**LIPYIGVQWSRASFD**								**EI, Post**					
45		**VQWSRASFDADTIQI**													
46		**SFDADTIQIAQPKLA**													
47		**IQIAQPKLASPVLNM**													
48	*C* *. pecorum* MOMP F	**KLASPVLNMTTWNPT**													
49	Variable Domain 4	**LNMTTWNPTLLGEAT**				**EIN**									
50		**NPTLLGEATSVNAGN**													**EI, Post**
51		**EATSVNAGNKYADVL**					**EIN**								
52		**AGNKYADVLQIVSLQ**						**EI, Post**							
53		**DVLQIVSLQINKLKS**				**EIN**									**EI, Post**
54		**SLQINKLKSRKACGV**													
55		**LKSRKACGVSMGATL**								**EI, Post**					
56	Conserved domain	**CGVSMGATLLDADKW**													
57		**ATLLDADKWAINGEL**													
58		**DKWAINGELRLINER**													
59		**GELRLINERAAHLSA**													
60		**NERAAHLSAQCRF**													
61		**LINERAAHLSAQCRF**													
62		**HALPVGNPAEPSLLI**													
63		**VWEGMSGDPCDPCAT**													
64		**DVPKQFTMGPIPTSS (VD1**)													
65		**TMGPIPTSSTSAADS (VD1**)													
66		**TSSTSAADSATPTER (VD1**)													
67	*C* *. pecorum*	**ADSATPTERNNAAY (VD1**)													
68	MOMP A Variable	**TPTERNNAAYGKHMH (VD1**)													
69	Domains 1, 2, 3 and 4	**GSSLEGKYPNANISN (VD2**)													
70		**LPLPTNAGTSNATDL (VD3**)													
71		**WNPTLLGERTSGTTF (VD4**)													
72	*C* *. pecorum*	**KTDVPKQFTMGTTPT (VD1**)													
73	MOMP G Variable	**GTTMGTTPTSAGAAA (VD1**)													
74	Domains 1, 2, 3 and 4	**TPTSAGAAATSNTSE (VD1**)													
75		**AAATSNTSEQRNNPA (VD1**)													
76		**TSEQRNNPAYGKHMH (VD1**)													
77		**GSTLNDMYPNANISN (VD2**)													
78		**LPLPTDAGTDAATGL (VD3**)													
79		**TLLGEATQVDNSNKF (VD4**)		**EIN**	**EIN**									**EIPre**	
80		**KTDVPKQFTMGPIPT (VD1**)						**EI, Post**							
81	*C* *. pecorum*	**QFTMGPIPTSSTSAE (VD1**)													
82	MOMP H Variable	**IPTSSTSAEDSATPT (VD1**)													
83	Domains 1, 2, 3 and 4	**SAEDSATPTERNNAA (VD1**)													**EI, Post**
84		**TPTERNNAAYHDAEW (VD1**)							**EI, Post**						
85		**TLLGQATQVDNSNKF (VD4**)		**EIN**	**EIN**									**EIPre**	
86		**MKKTLKSAFLSAAFF**													**EI, Post**
87	Conserved domain	**FLSAAFFAGDASLHA**								**EI, Post**					
88		**DASLHALPVGNPAEP**												**EI, Post**	

EIPre - epitopes already identified by the pre-immunised samples

EIN – epitopes identified upon natural infection

EI, Post - epitopes identified by the post-immunised samples in *Chlamydia* -free and infected koalas.

* All the animals were compared to the mean of four pre-immunised samples

^# $^ **@ Animal compared to its pre-immunised sample**

### Response profiles of plasma antibodies in koalas with natural *Chlamydia* infections

**Figure 3:**
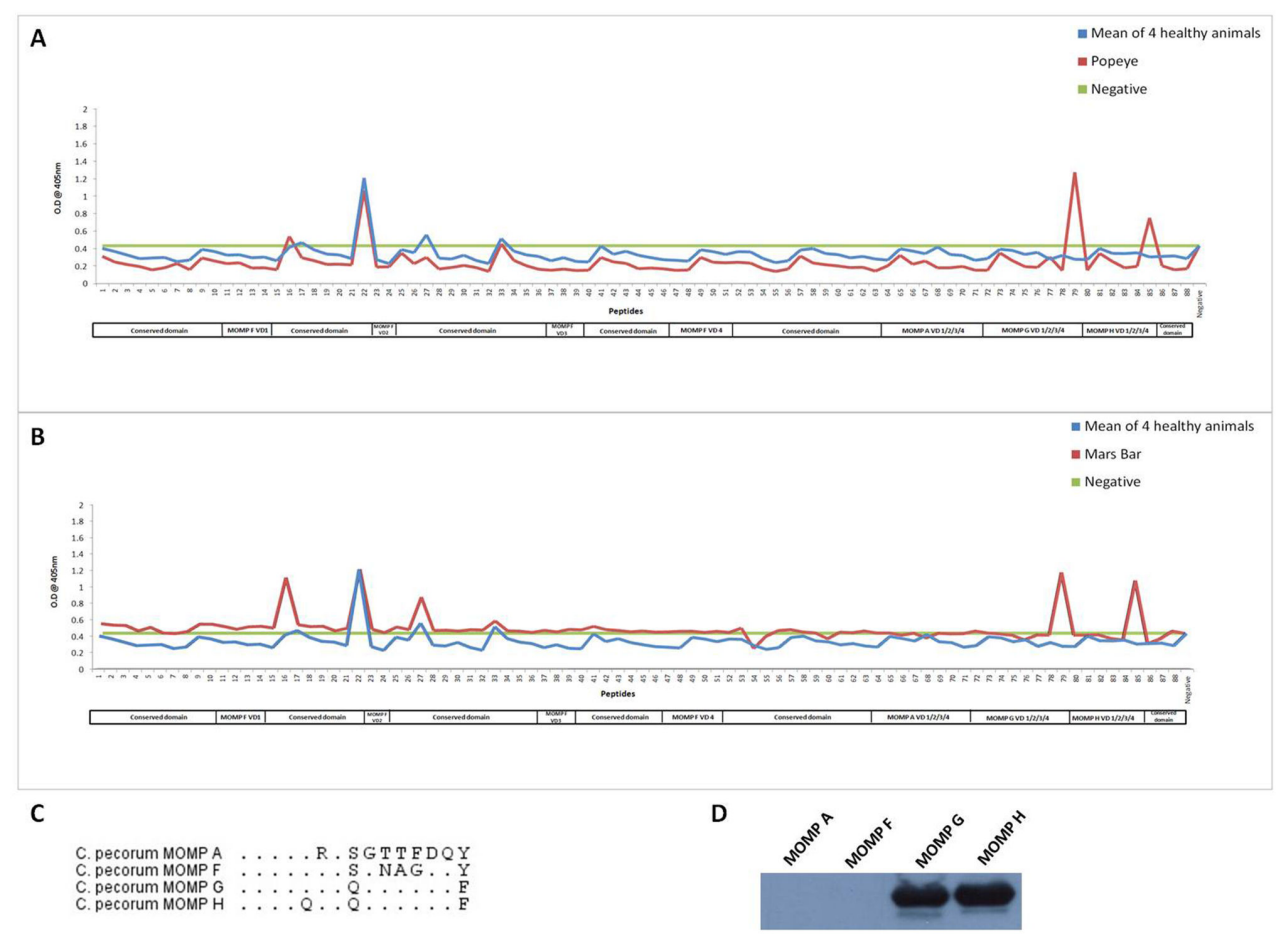
B cell epitope mapping across four MOMPs in naturally, *C* *. pecorum* genotype G infected koalas. Response profiles of two wild koalas naturally infected with *C* *. pecorum* G, Popeye (**A**) and Mars Bar (**B**) across MOMP peptide library and amino acid alignment of four MOMPs at VD4 epitope (**C**) (dots represent the consensus). Representative blot of plasma from the koalas infected with *C* *. pecorum* G against four recombinant full length MOMP proteins (**D**). The blue line indicates the mean of absorbance of four *Chlamydia* free animals. The red line indicates the *Chlamydia* infected animal. The green line indicates the background (Mean and two times standard deviation of negative controls).

Figure 3 shows the specificity of plasma antibodies produced in two animals [Popeye (Figure 3A) and Mars Bar (Figure 3B)] naturally infected with *C* *. pecorum* genotype G when compared to the response of four *Chlamydia* negative animals. Both animals had a similar response profile to the *Chlamydia* free animals for 84 out of 88 peptides, however, plasma from these two diseased animals reacted strongly with epitopes from variable domain four of MOMP G (298’-TLLG**E**ATQVDNSNKF;peptide 79) and the equivalent epitope in MOMP H (298’-TLLG**Q**ATQVDNSNKF;peptide 85), respectively. Interestingly, when this epitope of 15 amino acid length was aligned with all four MOMPs (Figure 3C), there are nine amino acid differences between MOMP G and MOMP A, eight amino acid differences between MOMP G and MOMP F but only one amino acid difference between MOMP G and MOMP H, which presumably explains the cross reactivity of plasma antibodies to MOMP G and MOMP H. Apart from the epitopes in the variable domains, plasma antibodies from Mars Bar also recognised two unique B cell epitopes in the conserved regions of MOMP (peptides 16 and 27), consistent with differences in immune responses in out-bred animals.

**Figure 4:**
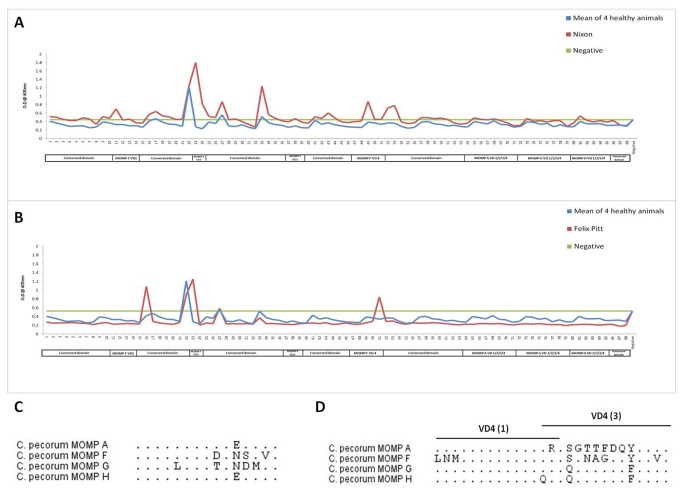
B cell epitope mapping across four MOMPs in naturally, *C* *. pecorum* genotype F infected koalas. Response profiles of two wild koalas naturally infected with *C* *. pecorum* F, Nixon (**A**) and Felix Pitt (**B**) across MOMP peptide library. Alignment of four MOMPs at an epitope at variable domain two (**C**), and two overlapping epitopes at variable domain four (**D**) (dots represent the consensus). The blue line indicates the mean of absorbance of four *Chlamydia* free animals. The red line indicates the *Chlamydia* infected animal. The green line indicates the background (Mean and two times standard deviation of negative controls).

**Figure 5:**
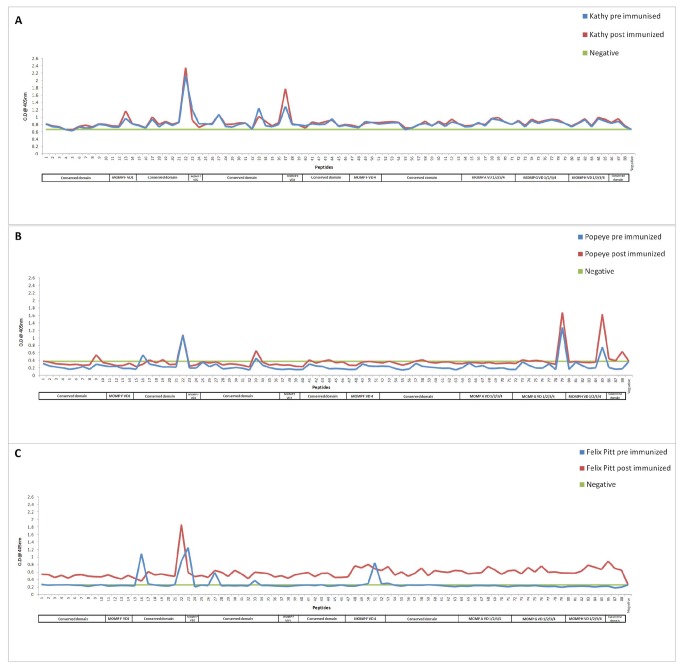
B cell epitope mapping across four MOMPs in *Chlamydia* infected koalas after administration with placebo or vaccine. Response profile of a *C* *. pecorum* F infected koala administered with a placebo (adjuvant only) across our MOMP peptide library (**A**). Response profiles of a naturally infected *C* *. pecorum* G koala immunized with recombinant MOMP G (**B**), and a naturally infected *C* *. pecorum* F koala immunized with recombinant MOMP G (**C**) across our MOMP peptide library. The blue line represents the absorbance of the pre-immunized sample. The red line represents the 20 weeks post-immunized sample. The green line indicates the background (Mean and two times standard deviation of negative controls).

Figure 4 shows the specificity of the plasma antibodies in two animals [Nixon (Figure 4A), and Felix Pitt (Figure 4B)] naturally infected with *C* *. pecorum* genotype F, again compared to the response of the four *Chlamydia* negative animals. Although both these wild koalas were infected with *C* *. pecorum* F, they had different responses to the MOMP peptides. Nixon (Figure 4A) produced antibodies to the three epitopes in the variable domains, SMGQAPSTNSPADSV (variable domain one; peptide 11), GLIGISGSDLNSKVP (variable domain two; peptide 23) and LNMTTWNPTLLGEAT (variable domain four; peptide 49) whereas Felix Pitt (Figure 4B) produced antibodies to epitopes GLIGISGSDLNSKVP (variable domain two; peptide 23) and EATSVNAGNKYADVL (variable domain four; peptide 51). For the epitope recognized by both animals in variable domain two (Figure 4C), we found four amino acid differences between MOMP F and the other three MOMPs. Interestingly, within the MOMP variable domain four (Figure 4D), both animals recognised two different epitopes VD4 (1) and VD4 (3), respectively, although the epitopes shared three amino acids between them. At VD4 (1) there are 3-5 amino acid differences between the various MOMPs and 5-8 amino acid differences between various MOMPs at VD4 (3). Another animal (Kathy), naturally infected with *C* *. pecorum* F also produced antibodies to epitopes DSVSLQERENPAYGK (variable domain one; peptide 13) and NQTLPLPITAGTATD (variable domain three; peptide 37) of MOMP F (Figure 5A).

### Evaluation of MOMP antibody responses in a *Chlamydia* infected animal administered with placebo (adjuvant only)

A *C* *. pecorum* F infected koala when vaccinated with a placebo (ISC) did not produce any antibodies to conserved domains, but retained a similar reactivity pattern to the pre-immunization sample (Figure 5A) by producing antibodies predominantly to the variable domains one and three of MOMP F.

### Enhancement of a broader repertoire of MOMP antibodies in naturally diseased animals following immunization with recombinant MOMP protein

Figure 5B illustrates the vaccine induced production of antibodies to various epitopes in a koala that was previously naturally infected with *C* *. pecorum* G. Interestingly, after immunization with recombinant MOMP G protein there was a significant production of antibodies against previously recognized epitopes (induced by natural infection) in variable domain four of MOMP G and H and additionally to the conserved domains (Table 2). Figure 5C shows the enhancement of antibodies in a *C* *. pecorum* F infected koala after immunization with MOMP G protein. The major epitopes recognized fall in the conserved domains as well as the variable domain four of MOMP F and variable domain one of MOMP H (Table 2).

## Discussion

Studies in various animal models (mice, guinea pigs and non-human primates) of *Chlamydia* infection, have suggested a significant requirement for antibody-mediated immune responses (neutralising antibodies) along with cellular immune responses (Th1) for an effective vaccine. Morrison et al. [20] demonstrated a significant role for B cells and CD4+ T cells but not CD8+ T cells, in resistance to *C. trachomatis* genital tract reinfection. In a follow up study, Morrison et al. [21] subsequently showed that antibody-mediated immune responses were critical in the resolution of secondary genital tract infection in mice but this resolution was independent of both CD4+ and CD8+ T cells. Hence, optimising the antibody response is essential for effective chlamydial vaccine development.

Our previous koala *Chlamydia* vaccine trials [17,18] have demonstrated strong vaccine induced humoral immune responses in healthy koalas as well as vaccine enhanced immune responses in naturally infected koalas, at both systemic and mucosal sites. One particularly encouraging aspect of these studies was the observation of cross-reactive antibodies in vaccinated healthy and diseased animals [17] that recognized MOMP from different *C* *. pecorum* strains. The functionality of these antibodies was demonstrated by their ability to cross-neutralize heterologous *C* *. pecorum* infections, *in vitro* [18].

In our current study, we used Pepscan methodology to investigate linear epitopes along the entire MOMP protein to determine the specificity of the antibody responses produced following, (a) natural live infection, (b) recombinant MOMP vaccination, (c) recombinant MOMP vaccination on top of natural live infection. The 15mer peptides (overlapping by 9 amino acids) covered the full length of koala *C* *. pecorum* MOMP F protein, as well as the variable domain regions for koala *C* *. pecorum* MOMPs A, G and H. When we evaluated immune sera from four naturally infected koalas, interestingly, only six linear epitopes were recognised. Five of these epitopes (peptides 23, 49, 51, 79 and 85) were located in the variable domains, while the other epitope (peptide 53) was in a conserved domain. These naturally recognised epitopes presumably represent the surface-exposed regions on intact chlamydial EBs/RBs that are recognised during a natural live infection. Of these six naturally recognised MOMP epitopes, none were recognised by all four koalas. One possible explanation for this could be due to major histocompatibility complex restriction in these out bred koalas [22]. We found that different epitopes were recognised in koalas infected with different *C* *. pecorum* genotypes (peptides 79 and 85 were recognised by *C* *. pecorum* G infected koalas only; peptides 23, 49 and 51 were recognised by *C* *. pecorum* F infected koalas only).

The *in silico* B cell prediction algorithm has predicted major epitopes in all four variable domains across *C* *. pecorum* MOMPs [18], however koalas naturally infected with *C* *. pecorum* G genotype produced antibodies only to the linear epitopes of variable domain four of MOMP G and MOMP H. This cross reactivity to MOMP G and MOMP H was similar to the immunoblot data we observed (Figure 3D). Interestingly, an alignment of the amino acids at this epitope, revealed only one amino acid difference between them i.e. Glutamic acid (E) in MOMP G and Glutamine (Q) in MOMP H (Figure 3C). Amino acids at variable domain four between MOMP G and H were more similar (93.3% consensus) when compared to the variable domain three (73.3% consensus), variable domain two (69.2% consensus) and variable domain one (62.5% consensus) regions, which explains the cross-reactivity of antibodies to MOMP G and H. Among the koalas infected naturally with *C* *. pecorum* F, one animal (Nixon) produced plasma antibodies to three epitopes at the variable domains of MOMP F (VD1, VD2 and VD4) whereas the other animal (Felix Pitt) produced antibodies to epitopes in two variable domains of MOMP F (VD2 and VD4). Both the animals (Nixon and Felix Pitt) shared a common epitope located in variable domain two. Even though there was 73.3% similarity between MOMP F and MOMP A at this linear epitope, and 66.6% between MOMP F and MOMP G/H, both koalas specifically produced antibodies only to MOMP F (Figure 4C). Surprisingly, although both targeting variable domain four of MOMP F, antibodies from each animal recognised two different overlapping epitopes within this domain (Figure 4D).

Pre-immunised plasma samples from four *Chlamydia* negative koalas, with the exception of Amity, did not recognise any of the epitopes except for peptide 22 (Figure 2). When we evaluated sera from four *Chlamydia* negative koalas that were immunised either with individual recombinant MOMP proteins (A, F and G) or a combination of two recombinant MOMP proteins (A and F), we observed an epitope response that was different to the response seen with naturally infected koalas (Figure 2) (Table 2). In these four immunised koalas, a total of 15 epitopes were recognised and eight of these epitopes were different to the epitopes recognised during a natural live infection. Only five of these vaccine induced epitope antibodies were in the variable domains of MOMP F and MOMP H, while 10 were in conserved domains. Furthermore, a koala immunised with both recombinant MOMPs (A and F) did not show any extra boosting effect to the conserved domains in particular, when compared to animals immunized with a single MOMP. This data strongly correlates with the observation of no significant difference in the cross-reactivity of antibodies post-vaccination between the individual and dual MOMP groups, as shown in Figure 2 and previously observed [18]. None of the *in silico* predicted B cell epitopes in the conserved domain [18] were recognized by the koalas; however antibodies to various other epitopes in conserved domains were elicited.

Early studies by Girjes et al. [23] reported *in vitro* neutralisation with sera from naturally infected koalas. However, in their study, less than 50% of the sera showed *in vitro* neutralisation activity and this varied between koalas. By comparison, the four koalas that were given a recombinant MOMP vaccine in our current study were all previously shown by Kollipara et al. [17] to produce *in vitro* neutralising antibodies. Although animals were immunized with single recombinant *C* *. pecorum* MOMPs, they still effectively cross neutralized the heterologous infections. Interestingly, there was no significant difference between individual and combined MOMP groups (MOMP A and MOMP F) in cross neutralization of *C* *. pecorum* infections. This is very encouraging from a vaccine development perspective, for the fact that seven of the recognised epitopes were in conserved domains and therefore conserved across all 13 *C* *. pecorum* MOMP genotypes (data not shown) that we currently have sequence information for, bodes well for wider cross neutralisation. It has also become evident from studies in naturally infected koalas that strong, long lasting protection from the neutralising antibodies does not result in natural infections [18]. Hence, an effective vaccine will need to improve on the existing natural immune response.

In the final aspect of our study, we evaluated the specificity of the response of koalas naturally infected with *C* *. pecorum* and then administered the recombinant MOMP vaccine. This is critical because, if the vaccine has to be delivered to wild populations, which already have 20-50% *C* *. pecorum* infections levels (Kollipara et al., 2013; unpublished data), it has to be effective in these groups. In the naturally infected vaccinated animals, a total of eight vaccine induced epitopes were recognised (Table 2). Out of eight epitopes, six were recognised in conserved domains and the remaining two in the variable domains (peptides 50 and 83).

In conclusion, although this study has dealt with the specificity of the polyclonal antibodies against MOMP in wild (outbred) koalas, known to have highly variable MHC Class II genes [22]. We have primarily focussed on expanding the findings (highly specific as well as cross-recognising humoral immune responses) from our previous studies [17,18]. In this study, we have shown, (a) specific MOMP B cell linear epitopes recognised in *C* *. pecorum* G and F genotype infected wild koalas and (b) MOMP linear epitopes responsible for the production of cross recognition antibodies in post-vaccinated healthy and diseased koalas. The findings from this study provides further insights into (a) screening various potential B cell epitopes using the Pepscan methodology in other chlamydial antigens, such as chlamydial heat shock protein (hsp60) [24] and thereby (b) developing diagnostic assays which aid in screening various healthy as well as diseased koalas. Using such findings as a proof of concept, a recent study [25] has identified new peptide antigens from *C. trachomatis* infected women and demonstrated the potential to develop an epitope-based serological diagnostic assay. Since both humoral and cell mediated responses are critical for a protective immune response against persistent chlamydial infections, our next step is to apply this screening assay to identify several potential T cell epitopes in healthy and diseased koalas.
